# Antioxidative and Chemopreventive Properties of *Vernonia amygdalina* and *Garcinia biflavonoid*

**DOI:** 10.3390/ijerph8062533

**Published:** 2011-06-23

**Authors:** Ebenezer O. Farombi, Olatunde Owoeye

**Affiliations:** 1 Drug Metabolism and Toxicology Research Laboratories, Department of Biochemistry, University of Ibadan, Ibadan, Nigeria; 2 Department of Anatomy, College of Medicine, University of Ibadan, Ibadan, Nigeria; E-Mail: oowoeye2001@yahoo.com

**Keywords:** *Garcinia kola*, *Vernonia amygdalina*, kolaviron, epivernodalol, antioxidants, chemoprevention

## Abstract

Recently, considerable attention has been focused on dietary and medicinal phytochemicals that inhibit, reverse or retard diseases caused by oxidative and inflammatory processes. *Vernonia amygdalina* is a perennial herb belonging to the Asteraceae family. Extracts of the plant have been used in various folk medicines as remedies against helminthic, protozoal and bacterial infections with scientific support for these claims. Phytochemicals such as saponins and alkaloids, terpenes, steroids, coumarins, flavonoids, phenolic acids, lignans, xanthones, anthraquinones, edotides and sesquiterpenes have been extracted and isolated from *Vernonia amygdalina.* These compounds elicit various biological effects including cancer chemoprevention. *Garcinia kola* (Guttiferae) seed, known as “bitter kola”, plays an important role in African ethnomedicine and traditional hospitality. It is used locally to treat illnesses like colds, bronchitis, bacterial and viral infections and liver diseases. A number of useful phytochemicals have been isolated from the seed and the most prominent of them is the *Garcinia* bioflavonoids mixture called kolaviron. It has well-defined structure and an array of biological activities including antioxidant, antidiabetic, antigenotoxic and hepatoprotective properties. The chemopreventive properties of *Vernonia amygdalina* and *Garcinia* biflavonoids have been attributed to their abilities to scavenge free radicals, induce detoxification, inhibit stress response proteins and interfere with DNA binding activities of some transcription factors.

## 1. Introduction

There has been a great deal of interest recently in the role of complementary and alternative medicines for the treatment of various acute and chronic diseases [1]. Of the various classes of phytochemicals, interest has focused on the anti-inflammatory and antioxidant properties of polyphenols found in various botanical agents. Plants vegetables and spices used in folk and traditional medicine have gained wide acceptance as one of the main sources of prophylactic and chemopreventive drug discovery and development [1].

Indeed, fruits and plants are rich sources of phenolic compounds and have been recognized to possess a wide range of properties including antioxidant, antibacterial, anti-inflammatory, hepatoprotective and anticarcinogenic actions. Many of the biological functions of flavonoids and phenolic compounds have been attributed to their free radical scavenging, metal ion chelating and antioxidant activities [2,3]. Antioxidant phenolic agents have been implicated in the mechanisms of chemoprevention which refers to the use chemical substances of natural origin or synthetic to reverse, retard or delay the multistage carcinogenesis process.

**Figure 1.**
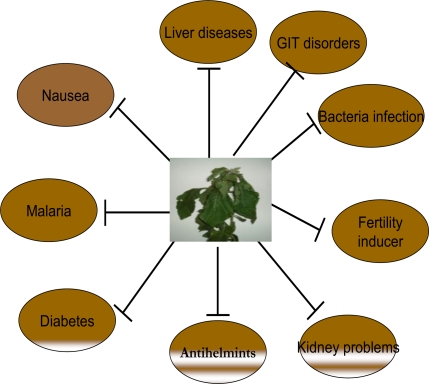
Traditional uses of *Vernonia amygdalina* (Bitter leaf).

*Vernonia amygdalina*, commonly known as bitter leaf, is a shrub that grows up to 3 meters high in the African tropics and other parts of Africa, particularly, Nigeria, Cameroon and Zimbabawe. It is reputed to have several health benefits. The organic fraction extracts of the plant was shown to possess cytotoxic effects towards human carcinoma cells of the nasopharynx [4]. It is effective against amoebic dysentery [5], gastrointestinal disorders [6], and has antimicrobial and antiparasitic activities [7,8]. Figure 1 depicts the various traditional uses of *Vernonia amygdalina.* The biologically-active compounds of *Vernonia amygdalina* are saponins and alkaloids [9], terpenes, steroids, coumarins, flavonoids, phenolic acids, lignans, xanthones and anthraquinone [10], edotides [11] and sesquiterpenes [4].

*Garcinia Kola* is a medium sized tree found in the moist forest and widely distributed throughout West and Central Africa. The edible nut is highly valued in these countries [12]. The seed, commonly known as bitter kola, is a masticatory and is a major kola substitute offered to guests at home and shared at social ceremonies. The seeds are used in folk medicine and in many herbal preparations for the treatment of ailments such as laryngitis, liver disorders, and bronchitis [13]. The biflavonoid mixture isolated from the seeds of *Garcinia kola* is known as kolaviron.

Various extracts of *Garcinia kola* have been found to elicit a number of biochemical properties including hepatoprotection, antidiabetic properties and antigenotoxic potentials. The focus of this review is on the antioxidative and chemopreventive properties of *Vernonia amygdalina* and *Garcinia* bioflavonoids from the seed of *Garcinia kola.*

## 2. *Vernonia amygdalina* (Asteraceae)

*Vernonia amygdalina*, a member of the Asteraceae family, is a small shrub that grows in the tropical Africa with petiolate leaf of about 6 mm diameter and elliptic shape. However, in contrast to the 1–3 meters described as the height of the tree by some authors [14–16], the species found around the Department of Wildlife and Fisheries, University of Ibadan, Nigeria are tall trees of greater than 6 metres in height [17]. It is commonly called *“bitter leaf”* because of its bitter taste. The bitterness can, however, be abated by boiling or by soaking the leaves in several changes of water. In Nigeria, it is known variously as “Ewuro” in Yoruba language, “Onugbu” in Igbo language, “Oriwo” in Bini language, “Ityuna” in Tiv, “Chusar doki or fatefate” in Hausa, while it is known as “Etidot” in Cross River State of Nigeria. The bitter taste is due to anti-nutritional factors such as alkaloids, saponins, tannins, and glycosides [18]. The leaves are used as green leafy vegetable and may be consumed either as a vegetable (leaves are macerated in soups) or aqueous extracts used as tonics for the treatment of various illnesses [14]. In the wild, chimpanzees have been observed to ingest the leaves when suffering from parasitic infections [19–21]. Many herbalists and native doctors in Africa recommend its aqueous extracts for their patients as treatment for varieties of ailments ranging from emesis, nausea, diabetes, loss of appetite, dysentery and other gastrointestinal tract problems to sexually transmitted diseases and diabetes mellitus among others [22] (Figure 1). Some of these and other uses have been verified experimentally and documented by various workers, thus providing scientific evidences to support many of these claimed health benefits [6,10,14,22–32].

### 2.1. Botanical Classification

The plant is scientifically classified as belonging to the Kingdom Plantae. It is an angiosperm, of the order Asterales, of the family Asteraceae, genus *Vernonia*, and *s*pecies *V. amygdalina.* The full binomial name is *Vernonia amygdalina* Del.

### 2.2. Compounds Isolated from *Vernonia amygdalina*

**Figure 2.**
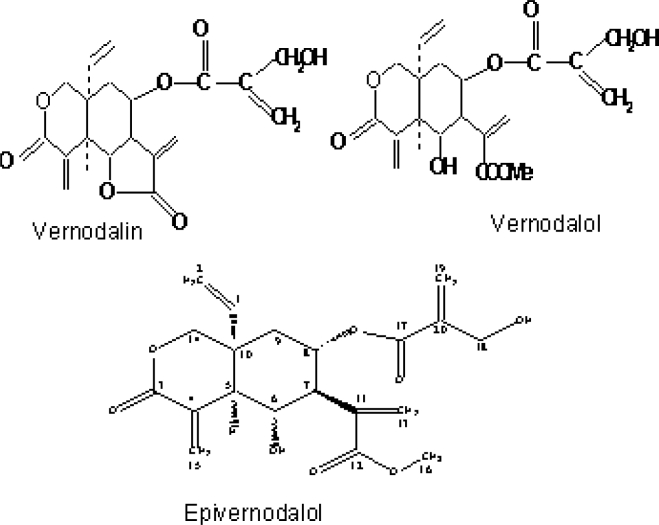
Some structures of compounds isolated from *Vernonia amygdalina*.

Several investigators have isolated and characterized a number of chemical compounds with potent biological activities from the leaves of *Vernonia amygdalina*. Some of the previously isolated constituents in *Vernonia amygdalina* Del. include: sesquiterpene lactones [10,11,14], flavonoids like luteolin, luteolin 7-O-glucosides and luteolin 7-O-glucuronide [33], steroid glycosides [14,34,35], and vernonioside A, B, A1, A2, A3, B2, B3 and A4 [14,34–36]. Edotides from the aqueous extract of the plant was characterized in [10]. Very recently, Owoeye *et al.* [37] isolated and characterized a sesquiterpene lactone, epivernodalol, another elemanolide from the dichloromethane fraction of *Vernonia amygdalina* (Figure 2). Koul *et al.* [38] isolated the compound epivernodalol from another species of the plant called *Vernonia lasiopus*. Some of these compounds are listed in Table 1.

### 2.3. Traditional Uses of *Vernonia amygdalina*

Leaves of this plant are used in Nigeria as a green vegetable or as a spice in soup, especially in the popular bitter-leaf soup. Such preparation includes freshly harvested leaves which are macerated with either cold or hot water to reduce the bitterness of the leaves to a desirable level. The latter are then added with other condiments for the soup while the water extract may be taken as a tonic to prevent certain illnesses. The leaves can be taken as an appetizer and the water extract as a digestive tonic [39]. These are largely consumed by the female Hausas in their belief that it makes them more sexually attractive.

In Northern Nigeria, it has been added to horse feed to provide a strengthening or fattening tonic called ‘Chusar Doki’ in Hausa [40]. The leaves have also been used in Ethiopia as hops in preparing tela beer [41]. The leaves are widely used for fevers and are known as a quinine–substitute in Nigeria and some other African countries [25,39,42,43]. The young leaves are used in folk medicine as antihelmintic, antimalarial, laxative/purgative, enema, expectorant, worm expeller and fertility inducer in subfertile women. Some wild chimpanzees in Tanzania had been observed to use this plant for the treatment of parasite related diseases [21,36]. Many herbalists and naturopathic doctors have recommended the aqueous extracts for their patients as treatment for emesis, nausea, diabetes, loss of appetite-induced abrosia, dysentery and other gastrointestinal tract problems.

### 2.4. Biological Activities of *Vernonia amygdalina*

#### 2.4.1. Antioxidant Activity

Iwalewa *et al.* [44] reported the antioxidant and cytoprotective activities of boiled, cold, and methanolic extracts of nine edible vegetables in Southwest Nigeria which were evaluated in the 1,1-diphenyl-2-picrylhydrazyl free radical assay and hemagglutination assay in bovine erythrocytes, respectively. While *Crassocephalum rubens* showed the highest antioxidant activity (56.5%), *Solanum americanum* and *Vernonia amygdalina* exhibited significant antioxidant activity. Subsequently, Iwalokun *et al.* [45] reported the antioxidant effects of an aqueous extract of *Vernonia amygdalina* leaves against acetaminophen-induced hepatotoxicity and oxidative stress in mice. Pre-administration of *Vernonia amygdalina* resulted in a dose-dependent reversal of acetaminophen-induced alterations of all the liver function parameters and suppressed acetaminophen-induced lipid peroxidation and oxidative stress. The study suggested that *Vernonia amygdalina* protected against acetaminophen-induced hepatic damage in mice by antioxidant mechanisms. The antioxidant mechanism of *Vernonia amygdalina* has been justified by the recent studies of Adesanoye and Farombi [46]. In this study, *Vernonia amygdalina* protected against carbon tetrachloride-induced liver injury by inducing antioxidant and phase 2 enzymes.

The antioxidant activity of *Vernonia amygdalina* has been attributed to the presence of flavonoids, as reported by Igile *et al.* [33]. Using spectroscopic techniques, the study had isolated and characterized the flavonoids occurring in *Vernonia amygdalina*. Three flavones were identified with chemical and spectroscopic techniques namely: luteolin, luteolin 7-*O-β*-glucuronoside, and luteolin 7-*O-β*-glucoside. Determination of the antioxidant activity of the three flavones had shown that luteolin showed greater activity than the other two. Since flavonoids are established as possessing antioxidant activity [47–52]. It can be speculated that the antioxidant properties of *Vernonia amygdalina* can be attributed to the presence of these flavonoids. The advantage of this antioxidant property has been revealed in neurotoxic studies since it has been established that flavonoids can traverse the blood brain barrier [53]. In this connection, Owoeye *et al.* [54] reported the neuroprotection of the cerebellum by the methanolic extract of *Vernonia amygdalina* leaves on the gamma-irradiated brain of Wistar rats.

#### 2.4.2. Chemopreventive Properties of *Vernonia amygdalina*

Kupchan *et al.* [11] reported the inhibitory activity of the chloroform extract of *Vernonia amygdalina in vitro* against cells derived from human carcinoma of the nasopharynx (KB) carried in tissue culture. Bioassay-guided fractionation of the extract yielded two compounds namely vernodalin (C_19_H_20_O_7_) and vernomygdin (C_19_H_25_O_7_), both of which had cytotoxic properties.

Izevbigie *et al.* [10,28] reported that the aqueous extract was a potent inhibitor of cultured human breast tumour cells (MCF-7) growth *in vitro.* This may imply tumour stabilization or preventive effects *in vivo.* Applying the reverse-phase chromatography method, fractions of *Vernonia amygdalina* extract was found to inhibit DNA synthesis. Much more important was the findings that the physiological concentrations of the water-soluble *Vernonia amygdalina* extract potently inhibited DNA synthesis in a concentration-dependent manner both in the presence and absence of serum. Further studies reported that treatment of cells with various concentrations (3–100 μg/mL of the same water-soluble extract potently inhibited cell growth of extracellular signal-regulated protein kinases 1 and 2 (ERKs 1/2) activities, DNA synthesis, and cell growth in a concentration-dependent manner. Izevbigie *et al.* [28] suggested that the ERK signaling pathways may be one or more of the intracellular targets for *Vernonia amygdalina* antineoplastic actions. Assessing the chemotherapeutic efficacy of V*ernonia amygdalina* (VA) leaf extracts as anti-cancer agent against human breast cancer *in vitro* using the MTT [3-(4,5-dimethylthiazol-2-yl)-2,5-diphenyltetrazolium bromide] and alkaline single cell gel electrophoresis (Comet) assays, respectively, Yedjou *et al.* [55] reported that *Vernonia amygdalina* treatment moderately (P < 0.05) reduced cellular viability and induced minimal DNA damage in MCF-7 cells. This provided evidence that *Vernonia amygdalina* extracts represent a DNA-damaging anti-cancer agent against breast cancer and its mechanisms of action functions, at least in part, through minimal DNA damage and moderate toxicity in tumors cells. In mechanistic terms studies have shown that *V. amygdalina* extract acts as a monofunctional inducer in MCF-7 breast cancer cells. Exposure of cells to low doses of *V. amygdalina* did not affect expression levels of CYP1A1/1A2 mRNA, but led to induction of microsomal epoxide hydrolase (mEH), thus supporting the chemotherapeutic potential of *Vernonia amygdalina* [56].

**Figure 3.**
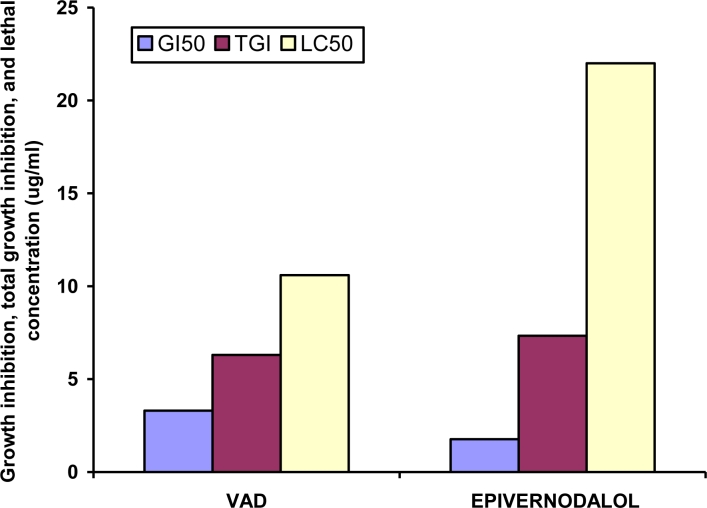
Each value represents the means ± SD of three independent experiments. **GI_50_** = Growth inhibition of 50% of the cells; **TGI** = Total growth inhibition; **LC_50_** = Lethal concentration of the compound/extract that kills 50% of the cells; **MEVA:** methanolic extract of *Vernonia amygdalina;* **VAP:** petroleum ether fraction; **VAD:** dichloromethane fraction. (Adapted from Owoeye *et al.* [37]).

Applying bioassay-guided fractionation of the methanolic extract of *V. amygdalina* leaves, Owoeye *et al.* [37], isolated and characterized a known sesquiterpene lactone compound, *epivernodalol* (C_20_H_24_O_8_) for the first time in this plant applying spectroscopic methods. *In vitro* growth inhibitory and cytotoxic evaluation of the extract, its fractions and epivernodalol against skin melanoma cell line (HT-144) was carried out by the Sulforhodamine B (SRB) assay. The results showed that the extract, the dichloromethane fraction, and epivernodalol (Table 2) were active against skin melanoma cell line (HT-144) (Figure 3).

The anticancer activity exhibited by epivernodalol could be explained by its being a sesquiterpene lactone. Rodriquez *et al.* [57] described the mechanism by which sesquiterpene lactones exhibit growth inhibition properties which depended on the structural configuration of the compound, namely the presence of an exocyclic methylene conjugated to the gamma-lactone, and the presence of a functional group such as hydroxyl or unsaturated ketone among other groups. A review of the epivernodalol molecule shows the attachment to its lactone an exocyclic methylene, and hydroxyl groups on carbons 6 and 18, in addition to the ketone groups attached to carbons 12 and 17 (Figure 2). These properties might have qualified epivernodalol to act as a growth inhibitor, as demonstrated in its activity against human skin cancer. The inhibitory action of sesquiterpene lactones results from the presence of highly electrophilic functional groups, which according to Rodriguez *et al.* [57], selectively alkylate by Michael-type addition to sulphydryl proteins, specifically thiol groups, in preference to other nucleophiles. Other methods by which *Vernonia amygdalina* extracts may suppress, delay, or kill cancerous cells include induction of apoptosis as determined in cell cultures and animal studies [58,59], enhanced chemotherapy sensitivity as *Vernonia amygdalina* extracts may render cancerous cells to be more sensitive to chemotherapy [59]. Further, down regulation of transcription factors have been implicated in the chemopreventive properties of VA. Thus suppression of metastasis of cancerous cells in the body by the inhibition of NF-κB, which is an anti-apoptotic transcription factors was demonstrated in animal studies [59]. The involvement of blood estrogen level in the etiology of estrogen receptor (ER) positive breast cancer has been widely reported and studies have shown positive correlations between blood estrogen levels and breast cancer risks [60]. Additional source of estrogen production in humans besides the ovary and adrenal gland is the conversion of testosterone to estrogen in a reaction catalyzed by aromatase. Therefore, compounds that inhibit aromatase activity are used for the treatment of breast cancer since there would be a reduction of estrogen level in the body by the suppression of aromatase activity [61].

## 3. *Garcinia kola* Heckel (Guttiferae)

### 3.1. Medicinal Uses of *Garcinia kola*

*Garcinia kola* is largely cultivated tree and highly valued in West and Central Africa for its edible nuts [12]. The seed, commonly known, as ‘bitter kola’ is eaten by many and it is culturally acceptable in Nigeria. Extracts of the plant have been employed in the African herbal medicine for the treatment of ailments such as laryngitis, liver diseases [13] cough and hoarness of voice [62].

The seed is employed as a general tonic and it is believed to have aphrodisiac properties. The roots and stems are cut into short chew-sticks used for cleaning teeth. *Garcinia kola* extract among ten common Nigerian chewing sticks examined for antibacterial properties, displayed good activity [24]. *Garcinia kola* seeds have also been indicated in the traditional African medicine in the treatment of inflammatory disorders. Extracts of the various parts of the plants have been employed in the treatment of laryngitis [12] and liver disease [13]. Extracts of the seeds gave a remarkable improvement of liver function in patients with chronic hepatitis and cholangitis after treatment for 14 days at a Nigeria herbal home [13].

### 3.2. Biological Properties of *Garcinia kola*

**Figure 4.**
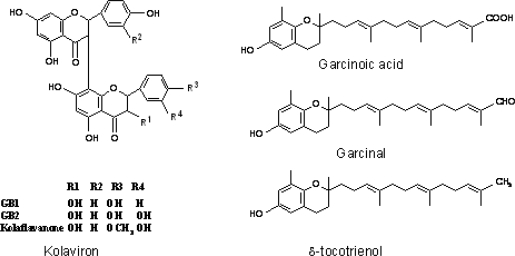
Chemical Structure of compounds isolated from *Garcinia kola.*

**Figure 5.**
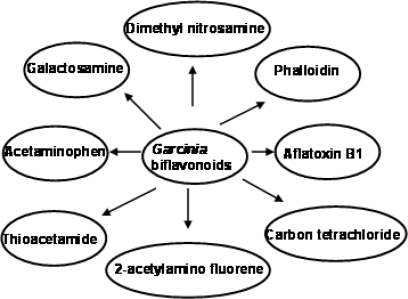
Hepatoprotective effects of *Garcinia* biflavonoids on selected hepatotoxins.

*Garcinia kola* and its extracts have been shown to elicit a number of biological activities in various experimental models. Rats chronically fed diets containing powdered seeds of *Garcinia kola* at the level of 10% w/w for 6 weeks displayed marked inhibition of gastrointestinal motility, protection against castor oil induced diarrhea and prolonged pentobarbital sleeping time [63]. Garcinia biflavonoid complex, also known as kolaviron (Figure 4) have been shown to elicit hypoglycaemic effect on both fasted normoglycemic and alloxan-diabetic rabbits [64]. Also the complex inhibited rat lens aldose reductase activity, an enzyme found in the lens and other tissues which have been implicated in many diabetic complications such as neuropathy and retinopathy [64–66]. The biflavanones of *Garcinia kola* have been shown to be pharmacologically active with several pharmacokinetic advantages over simple monomeric flavonoids as they survived first-pass metabolism which inactivates most flavonoids [67]. In addition to the above notable biological effects of Garcinia biflavonoid complex, studies have also shown the ability of this complex to protect against hepatotoxicity induced by phalloidin, amanita, 2-acetylaminofluorene, carbon tetrachloirde, paracetamol, aflatoxin, dimethyl nitrosamine in rodents [68–71] (Figure 5).

### 3.3. Chemical Composition of *Garcinia kola*

Studies involving bioassay-guided fractionation of *Garcinia kola* seed have yielded complex mixtures of phenolic compounds, triterpenes and benzophenones. Aplin *et al.* [72] isolated cycloartenol and its 24-methylene derivatives from the petroleum spirit extract of the seeds. The ethyl acetate-soluble fraction of the acetone extract contains C-3/8″-link biflavanone GB1, GB2, GB1a and kolaflavanone [73]. These biflavanones and their glycosides were also isolated from the stem back [13]. The ether soluble fraction of the alcoholic extract yielded apigenin-5,7,4′-trimethyl ether, apigenin-4′-methylether, fisetin, amento-flavone, kolaflavanone and GB1 [13]. Benzophenone and kolanone with potent antimicrobial properties were isolated from the light petroleum extract of *Garcinia kola* seeds [74]. Subsequently, Garcinia biflavonoid complex containing GB1, GB2 and kolaflavanone, a deffated fraction of alcoholic extract of *Garcinia kola* seeds was isolated [75]. This fraction is also popularly referred to as kolaviron. Recently, GB1 one of the major components of *Garcinia* biflavonoid was isolated from the roots of *Garcinia kola*. It showed inhibitory effects against methicilin-resistant *Staphylococcus aureus* (MRSA) and vancomycin-resistant *enteroccoci* (VRE). Terashima *et al.* [76], also reported two new chromanols, garcinoic acid and garcinal, together with δ-tocotrienol isolated from *Garcinia kola* seeds (Figure 4).

### 3.4. Antioxidant and Radical Scavenging Effects of *Garcinia* Biflavonoid (kolaviron)

**Figure 6.**
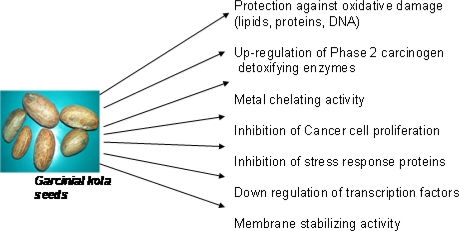
Biochemical mechanisms underlying chemopreventive properties of *Garcinia kola* derived-bioflavonoids.

A plethora of mechanisms have been proposed and implicated in chemoprevention of various degenerative diseases including cancer and antioxidant actions have been suggested as one of the prominent mechanism. Chemoprevention by natural products against oxidative damage and chemical carcinogens may therefore be related to their intrinsic antioxidant properties. Biochemical mechanisms underlying chemopreventive properties of *Garcinia kola* derived- bioflavonoids are shown in Figure 6.

**Figure 7.**
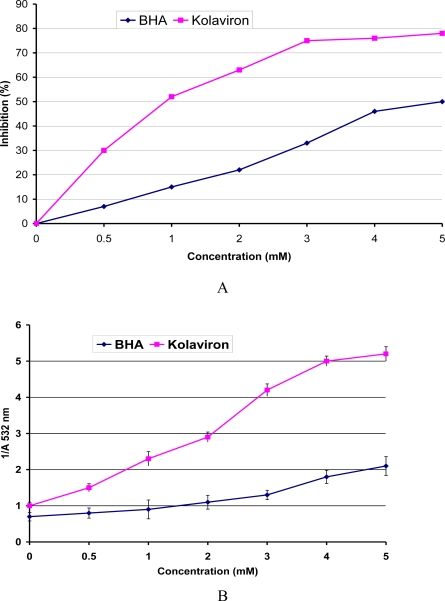
**(A)** Inhibition of deoxyribose oxidation by kolaviron and BHA. **(B)** A plot of Reciprocal Absorbance of TBARS at 532 nm against concentration of kolaviron and BHA for the Calculation of the rate constants. Values are means SD for three Experiments. (Adapted from Farombi *et al.* [49]).

The antioxidant and scavenging activity of Garcinia biflavonoid complex has been investigated in a range of established *in vitro* assays involving reactive oxygen species. The study showed that kolaviron elicited significant reducing power and a dose-dependent inhibition of oxidation of linoleic acid [49]. Kolaviron inhibited H_2_O_2_, and was more effective than the standard antioxidants BHA and β-carotene and equivalent in power to α-tocopherol. Kolaviron also significantly scavenged superoxide generated by phenazine methosulfate NADH. Furthermore, kolaviron scavenged hydroxyl radicals as revealed by significant inhibition of the oxidation of deoxyribose (Figure 7). Hydroxyl radical is highly reactive oxygen centered radical, which attacks all proteins, DNA, polyunsaturated fatty acids in membranes and almost any biological molecule it touches [74,77]. The inhibitory activity of kolaviron in deoxyribose assay may relate directly to prevention of the propagation of the process of lipid peroxidation and modulation of other biomarkers of oxidative stress in animal model [68,69]. Farombi *et al.* [78] related the chemoprotective effect of kolaviron to its *in vivo* antioxidant effects. Thus, kolaviron reduced background levels of protein oxidation marker (2-aminoadipic semialdehyde) in plasma and liver and γ-glutamyl semialdehyde (GGS) as well as malondialdehyde in liver [78]. In addition, kolaviron reduced damage to proteins and lipids induced by Fe^3+^/EDTA/ascorbate mixtures *ex vivo* [79].

Furthermore, kolaviron dose dependently inhibited the intracellular ROS production induced by H_2_O_2_ in HepG2 cells detected as 2′7′-dichlorofluorescein diacetate (DCF) fluorescence [80,81]. Thus the ability of kolaviron to act as antioxidant in this cell underscores its role as an antioxidant and its potential role in the chemoprevention of chemically-induced genotoxicity.

#### 3.4.1. Effects of *Garcinia* Biflavonoid on LDL Oxidation and Biomarkers of Oxidative Stress

Oxidation of low-density lipoprotein (LDL) is generally believed to promote atherosclerosis, primarily by leading to an increased uptake of oxidized LDL (ox-LDL) by macrophages and subsequent foam cell formation [82]. Therefore, inhibition or reduction of the process of LDL oxidation may delay the progression of atherosclerosis. Several antioxidants such as vitamin E and C and flavonoids have been shown to reduce the *in vitro* oxidation of LDL. Lipoprotein resistance to copper-induced oxidation was highly improved in rats treated with *Garcinia* biflavonoid (100 mg/kg) for 7 days as demonstrated by significant increase in lag time, a decrease in area under the curve (AUC) and slope of propagation [82]. Conjugated diene formed after 240 min of lipoprotein oxidation and malondialdehyde concentrations were markedly decreased in the *Garcinia* biflavonoid treated rats with attendant significant increase in the total antioxidant activity compared to control. In addition, *Garcinia* biflavonoid (10–60 μM) inhibited the Cu^2+^-induced oxidation of rat serum lipoprotein in a concentration-dependent manner and at the highest dose tested (90 μM) elicited a significant chelating effect on Fe^2+^. Furthermore, *Garcinia* biflavonoid effectively prevented microsomal lipid peroxidation induced by iron/ascorbate in a concentration dependent manner [82]. The data demonstrate that *Garcinia* biflavonoid protected against the oxidation of lipoprotein presumably by mechanisms involving metal chelation and antioxidant activity and as such might be of importance in relation to the development of atherosclerosis.

#### 3.4.2. Effects of Kolaviron on Oxidative DNA Damage

Many chemicals including hydrogen peroxide are known to generate DNA damage through an oxygen radical mechanism and can induce chromosomal aberrations, gene mutations and DNA strand breaks [83]. Flavonoid compounds possessing antioxidant and antiradical activities are capable of quenching free radicals, which may promote mutations and DNA damage. *Garcinia* biflavonoid at concentrations between 30–90 μM decreased H_2_O_2_-induced DNA strand breaks and oxidized purine (formamidopyrimidine glycosylase (FPG) and pyrimidine (Endonuclease III (ENDO 111) sites) bases in both human lymphocytes and rat liver cells [79]. Furthermore, in lymphocytes incubated with Fe^3+^/GSH, Fe^3+^ was reduced to Fe^2+^ by GSH initiating a free radical generating reaction which induced increases in strand breaks, ENDO III and FPG sensitive sites. However, *Garcinia* biflavonoid at 30 and 90 μM significantly attenuated GSH/Fe^3+^-induced strand breaks as well as base oxidation [79]. In addition, *Garcinia* biflavonoid *ex vivo* protected rat liver cells against H_2_O_2_-induced formation of strand breaks, ENDO 111, and FPG sensitive sites. Farombi *et al.* [78] further demonstrated the protective effect of *Garcinia* biflavonoid *in vivo* against oxidative damage to DNA in rat liver. These findings suggest that *Garcinia* biflavonoid exhibits protective effects against oxidative damage to DNA via scavenging of free radicals and iron binding.

### 3.5. Chemopreventive Properties of *Garcinia* Biflavonoid

#### 3.5.1. Antigenotoxic Effect of *Garcinia* Biflavonoid

We investigated the chemopreventive effects of kolaviron, on aflatoxin B1 (AFB1), a potent hepatocarcinogen in experimental animals [84] and human carcinogen [85]. Investigations of the chemopreventive effects of kolaviron on AFBI induced genotoxicity in rats showed that co-treatment of rats with kolaviron after AFB1 administration inhibited the induction of micronucleated polychromatic erythrocytes. In addition, kolaviron inhibited AFB1-induction of markers of hepatic oxidative damage [70]. Furthermore, administration of rats with kolaviron alone resulted in significant elevation in the activities of phase 2 enzymes—glutathione *S*-transferase, uridyl glucuronosyl transferase and NADH:quinone oxidoreductase—by 2.45, 1.62 and1.38-fold, respectively.

**Figure 8.**
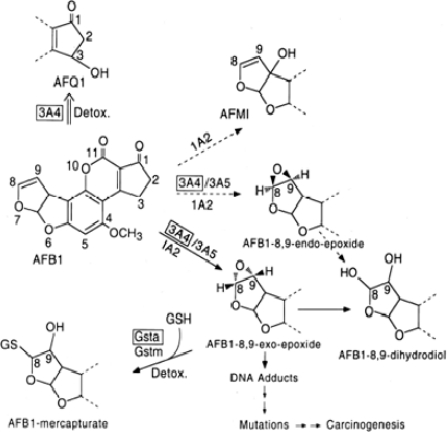
Proposed mechanism of aflatoxin B1-induced DNA damage (Adapted from Nwankwo *et al.* [80]).

Nwankwo *et al.* [80] reported the ability of *Garcinia* biflavonoid to inhibit aflatoxin B_1_ induced genotoxicity in HepG2 cell. AFB1 requires microsomal oxidation to the reactive AFB1-8,9, epoxide (AFBO) to exert its hepatocarcinogenic effects (Figure 8). The *exo* epoxide predominates and it is also more genotoxic than the *endo* form which is also produced [84]. The CYP isozymes involved in the metabolism of AFB1 are the 1A2, 3A4 and 3A5 [86]. 1A2 metabolism yields mostly activated epoxide metabolite and detoxifies product aflatoxin M1 (AFM1) [84]. The 3A4, which is readily inducible in adult human liver [86], is known to yield a higher ratio of AFQ1 to epoxide metabolite than is the case for 1A2 at higher substrate concentration. AFBO is detoxified by the activity of GST and GSTα1-1 subclass is reputed to be the most abundant form in human liver and kidney and it is inducible by a variety of xenobiotics in HepG2 cells [87]. In the study of Nwankwo *et al.* [80], kolaviron specifically induced CYP 3A4. Furthermore, GST isozyme α-1 and α-2.2 were also induced for their messages as determined by Reverse transcription polymerase chain relation (RT-PCR) and northern blot analysis and GST α protein by western blotting. These findings explain at least in part the possible molecular mechanisms of action of antigenotoxic properties of kolaviron and is in agreement with previous reports [69,70]. The findings underscore the role of induction of phase 2 detoxifying enzymes as a mechanism of its hepatoprotective action.

#### 3.5.2. Effect of *Garcinia* Biflavonoid on Stress Response Proteins and Transcription Factors

Like other early-response gene products, COX-2 can be induced rapidly and transiently by proinflammatory mediators, endotoxins as well as carcinogens [80,88]. Inducible nitric oxide synthase (iNOS) is another inducible enzyme that causes the overproduction of nitric oxide during inflammation and tumor development [89,90]. Therefore, suppression of the induction and activity of COX-2 and/or iNOS has been considered a new paradigm in cancer chemoprevention in several organs [90,91].

**Figure 9.**
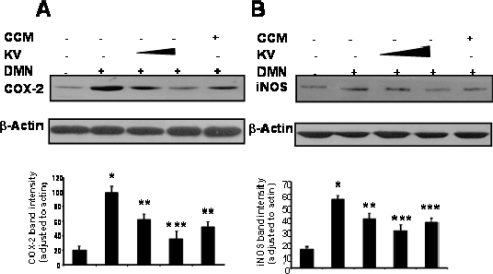
Inhibitory effect of kolaviron and curcumin on DMN induced COX-2 expression in rat liver (Adapted from Farombi *et al.* [71]). Quantification of COX-2 (A) and iNOS (B) immunoblots were normalized to that of actin followed by statistical analysis of relative image density in comparison to control. Lane 1, control; lane 2, DMN treated; lane 3, kolaviron (100 mg/kg) plus DMN; lane 4, kolaviron (200 mg/kg) plus DMN; lane 5, curcumin (200 mg/kg) plus DMN. * P < 0.001 compared with lane 1; ** P < 0.05 compare with lane 2; *** P < 0.01 compared with lane 2.

**Figure 10.**
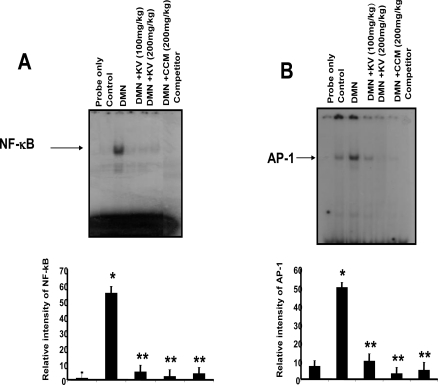
Inhibitory effects of kolaviron and curcumin on DMN-induced activation of NF-κB **(A)** and AP-1 **(B)** in rat liver (Adapted from Farombi *et al.* [71]). Lane 1, free probe; lane 2, control; lane 3, DMN alone; lane 4, kolaviron (100 mg/kg) plus DMN; lane 5, kolaviron (200 mg/kg) plus DMN; lane 6, curcumin (200 mg/kg) plus DMN; lane 7, competitor. * P < 0.001 compared with lane 2; ** P < 0.001 compared with lane 3.

Recent findings on the molecular mechanisms underlying hepatoprotective action of kolaviron indicate that it can suppress certain proinflammatory genes whose expression have been shown to be regulated by transcription factors. Kolaviron abolished the expression of COX-2 and iNOS proteins in dimethyl nitrosamine (DMN)-treated rat liver (Figure 9) suggesting that kolaviron may be important not only in alleviating liver inflammation but probably for the prevention of liver cancer [71]. NF-κB is ubiquitous transcription factor that resides in the cytoplasm as heterodimer consisting of p50, p65 and IκBα subunits, Upon activation, it translocates to the nucleus where it induces gene transcription [92]. On activation, IκBα undergoes phosphorylation and ubiquitination-dependent degradation by 26S proteosomes, thus exposing nuclear localization signal on p50–p65 heterodimer, leading to its nuclear translocation. In the nucleus, it binds to a specific consensus sequence in the DNA (5,-GGGACTTTC-3′) called κB binding site. On activation, NF-κB induces expression of more than 200 genes including COX-2. The transcription factors NF-κB and AP-1 have been reported to be key regulators of inflammatory protein such as COX-2 and iNOS [92]. Pretreatment of rats with kolaviron abrogated the DNA binding activity of NF-κB and AP-1 induced by DMN [71] (Figure 10).

**Figure 11.**
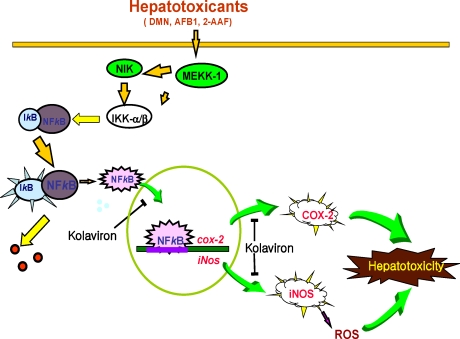
Proposed molecular mechanism of hepatoprotective properties of kolaviron (Adapted from Farombi [93]).

Agents that can suppress NF-κB and AP-1 have the potential of preventing the onset of cancer [90]. Therefore the inhibition of DMN-mediated DNA binding of these transcription factors and expression of proinflammatory proteins by kolaviron partly explains the molecular basis of the hepatoprotective effect of kolaviron in drug-induced hepatotoxicity and possibly hepatocarcinogenesis and may be an additional chemotherapeutic application of the naturally occurring flavonoid. The proposed underlying molecular mechanism of hepatoprotective activity of kolaviron [93] is depicted in Figure 11.

#### 3.5.3. Antiproliferative Potentials of *Garcinia* Biflavonoid

*Garcinia* biflavonoids have been studied for its effects on survival of colon adenoma (LT97) and carcinoma-derived (HT29) cell lines. LT 97 cells isolated from micro-adenoma of patient with familiar adenoma polyposis are cells of an early colon adenoma in the premalignant stage of tumor development whereas HT29 cell line was established by Fogh and Trempe from a colon adenocarcinoma of a Caucasian female [94]. Preliminary [95] demonstrate that *Garcinia* biflavonoid complex suppressed the growth and survival of both adenoma (LT97) and carcinoma cells (HT29). Flavonoid and polyphenolic compounds have been shown to elicit antiproliferative properties by inhibiting the growth of these cells [96]. The data showed that adenoma cells were more sensitive than the transformed carcinoma cells suggesting a higher chemopreventive potential of *Garcinia* biflavonoid mixture in the preneoplastic lesion than the carcinoma. The results showed that the antiproliferative effect of *Garcinia* biflavonoids may qualify the compounds as chemopreventive agent against colon carcinogenesis.

## 4. Conclusions and Future Direction

A number of chemical compounds including edotides, and sesquiterpene lactones have been isolated form the leaf of *Vernonia amygdalina*. Similarly, the bioflavonoid complex otherwise known as kolavion have been characterized from the seed of *Garcinia kola*. These compounds elicit remarkable antioxidant and chemopreventive properties in cell cultures and rodent models. Prominent among the biochemical and molecular mechanisms of action of these compounds are elevation of phase II enzymes, inhibition of cell proliferation and suppression of pro-inflammatory mediators. These mechanisms play pivotal role in chemoprevention which appears to be a more pragmatic and rational approach to prevention of cancer. Cancer chemoprevention has received a phenomenal attention in the last three decades by several investigators with focus on evaluating the efficacy of well defined phytochemicals from dietary substances and plant origin in various experimental models. There is also the need to source for new and novel chemopreventive agents from natural origin. In this review, the chemopreventive properties of novel compounds from *Vernonia amygdalina* and *Garcinia kola* have been presented. Their striking underlying and molecular mechanisms support the idea that supplements of these compounds may be considered in strategies of using natural products in cancer prevention and therapy. Necessary long-term clinical trials are therefore warranted in order to showcase potential benefits of *Vernonia amygdalina* and *Garcinia kola*-derived phytochemicals as chemopreventive agents. Also important is bioavailability of the compounds in *Vernonia amygdalina* and *Garcinia kola*. However, the biflavanones of *Garcinia* are pharmacologically active with several pharmacokinetic advantages over simple monomeric flavonoids; for instance, the bifavonoids have been shown to survive first-pass metabolism which inactivates most flavonoids and they have been proved to possess very high thera-peutic potentials [67].

## Figures and Tables

**Table 1.** Bioactive compounds isolated from *Vernonia amygdalina.*

**Name of Compound**	**Class of Compound**	**Author (s)**
Vernodalin	Sesquiterpene lactone	Kupchan *et al.* (1969) [4]
Vernomygdin	Sesquiterpene lactone	Kupchan *et al.* (1969) [4]
Vernoniosides A_1_, A_2_, A_3_, B_1_	Steroid Glucosides	Jisaka *et al.* (1992) [34]
Vernoniosides A_4_, B_2_, B_3_	Steroid Glucosides	Jisaka *et al.* (1993) [35]
Vernoniosides D and E	Steroid glycosides	Igile *et al.* (1995) [33]
Vernodalol	Sesquiterpene lactone	Ganjian *et al.* 1983 [23]; Erasto *et al.* 2006 [16]
Epivernodalol	Sesquiterpene lactone	Owoeye *et al.* (2010) [37]

**Table 2.** Activity of *Vernonia amygdalina* Del. extract, fractions, Epivernodalol and doxorubicin against HT-144 (skin melanoma) cell line.

**Code**	**GI_50_ (μg/mL)**	**TGI (μg/mL)**	**LC_50_ (μg/mL)**
MEVA (extract)	86 ± 1.3	141.3 ± 4.7	199 ± 10.8
VAD (fraction)	3.3 ± 0.3**	6.3 ± 0.3**	10.6 ± 0.8**
VAP (fraction)	9.7 ± 2.3*	21.2 ± 5.4*	37.7 ± 4.4*
Epivernodalol	1.76 ± 0.3**	7.33 ± 0.55**	22 ± 1.2**
Doxorubicin	0.01 ± 0	0.07 ± 0.03	0.48 ± 0.1

Each value represents the means ± SD of three independent experiments.

* Significantly different from MEVA (p < 0.05).

** Significantly different from MEVA (p < 0.01). **GI_50_** = Growth inhibition of 50% of the cells; **TGI** = Total growth inhibition. **LC_50_** = Lethal concentration of the compound/extract that kills 50% of the cells. **MEVA:** methanolic extract of *Vernonia amygdalina;* **VAP:** petroleum ether fraction; **VAD:** dichloromethane fraction. (Adapted from Owoeye *et al.* [37]).
